# Supplementary material for: A high-resolution mRNA expression time course of embryonic development in zebrafish
Source: eLife. 2017 Nov 16;6:e30860. doi: 10.7554/eLife.30860 (PMC5690287; doi:10.7554/eLife.30860)
Supplement: Supplementary file 6. [file elife-30860-supp6.zip › biolayout-clusters-files/Cluster176-genes.html]

Cluster176


# Cluster176: Genes

| Ensembl ID | Gene Name | Chr | Start | End | Biotype |
| --- | --- | --- | --- | --- | --- |
| ENSDARG00000078123 | SLITRK3 (1 of many) | 18 | 2146234 | 2148849 | protein\_coding |
| ENSDARG00000104085 | abcd3a | 24 | 31373429 | 31394876 | protein\_coding |
| ENSDARG00000071150 | cica | 19 | 6166707 | 6215771 | protein\_coding |
| ENSDARG00000102216 | dlg1l | 2 | 5059548 | 5234147 | protein\_coding |
| ENSDARG00000103840 | lrrc8db | 6 | 25071499 | 25081203 | protein\_coding |
| ENSDARG00000100170 | pam | 10 | 5688306 | 5786096 | protein\_coding |
| ENSDARG00000005423 | pgam1a | 13 | 31262742 | 31267537 | protein\_coding |
| ENSDARG00000090721 | prdm2a | 8 | 26840500 | 26845298 | protein\_coding |
